# Modified tension band wiring of patellar fracture as a technique to minimize postoperative complications: A case report

**DOI:** 10.1097/MD.0000000000019576

**Published:** 2020-03-20

**Authors:** Tiecheng Yu, Zhendong Wu, Sayid Omar Mohamed, Weina Ju, Xiuxin Liu, Baochang Qi

**Affiliations:** a Department of Orthopedic Traumatology, The First Hospital of Jilin University; b Department of Burns, The First Hospital of Jilin University, Changchun; c Department of Orthopedic, Jazeera University Hospital, Mogadishu, Somalia; d Department of Neurology, The First Hospital of Jilin University, Changchun; e Department of Orthopedic Traumatology, The Sixth Affiliated Hospital of Xinjiang Medical University, China.

**Keywords:** double bundle wire stitching, patellar fracture, tension band wiring

## Abstract

**Rationale:**

Tension band wiring is the most widely accepted technique for the treatment of patellar fractures but the technique is associated with common complications like wire migration, prominence, and breakage. To reduce these complications, we developed and propose a modified technique that has a superior biomechanical strength and a potential to reduce such postoperative complications.

**Patient concerns:**

The patient presented with pain and mild swelling in his left knee after he slipped on the floor and fell on his left knee. He has no significant past medical or surgical history. The patient took the tension band wiring as the first choice because of the wide acceptance. But he worried about the complications.

**Diagnoses:**

X-ray showed a transverse fracture of the left patella with an inferior pole occult fracture.

**Interventions:**

The patient was operated with a modified technique of the classic tension band wiring for patellar fractures. In our 4-step procedure, double tension cerclage wires were wrapped under the exposed ends of the Kirschner wires (K-wires) and the tendons in figure-of-8 fashion. The aim was to increase the biomechanical strength so that when one of the tension wires fail, the other one can hold the fragments together.

**Outcomes:**

The patient recovered very well and without any complications. The patient was followed-up for 1 year and the fracture has united very well, with satisfying knee range of motion.

**Lessons:**

From this case study, we can detect the biomechanical advantages of our technique which can increase the stability of the fracture and that allows early functional exercise and additionally the micromotion at the fracture site has a beneficial effect of fracture union. Based on the perfect outcomes, our technique is worthy of clinical application.

## 1 Introduction

Patella is the largest sesamoid bone in the whole body.^[1]^ Fractures of the patella comprise about 1% of all fractures in adults with the absolute indication for operative treatment being an extensor mechanism disruption.^[2]^ Several surgical fixation methods have been reported in the literature for patellar fracture, including tension band, plates and screws, cerclage wiring, external fixation, and so on.^[3]^ And the tension band technique has been widely accepted as the golden standard for the treatment of patellar fractures because of its biomechanical advantages. But tension band wiring is not without complications. Smith et al^[4]^ reported 8% to 22% of hardware failure in patients treated with this technique, including hardware migration, prominence, and breakage. Although surgeons have tried numerous methods to avoid these complications, the optimal solution is still lacking. To minimize the complications, we developed a modified tension band wiring technique which involves double wire stitching in figure-of-8 pattern.

## 2 Surgical technique

The basic steps of the procedure including patient positioning, tourniquet use, surgical approach, and closure are all similar with the classic tension band wiring technique. No special instruments are required for our new technique; only the routine orthopedic trauma instruments set and 2 monofilament stainless steel wires with a needle whose diameter is 18-gauge (Covidien Medical Products (Shanghai) Manufacturing L.L.C , Shanghai , China) are needed. The procedure involves the following 4 key steps:

1. Reduce the fracture anatomically and hold it with a pointed reduction forceps. Then reduction is confirmed with fluoroscopy.
2. Inserting 2 parallel Kirschner wires (K-wires), without penetrating the articular cortex.
3. This step is the key step, 2 monofilament stainless steel wires with a needle whose diameter is 18-gauge (COVIDIEN) are need. And we refer this step as double bundle wire stitching. Stitch the first monofilament stainless steel wire as close as possible to the angle between patella and the exposed ends of K-wire under the tendon, using a figure-of-8 pattern in a vertical orientation. For the whole course, the wire should be wrapped as close as possible to the bone without soft tissue interposition both in the proximal and distal fragments. Then, the wires are tightened in a figure-of-8 fashion and knotting the wires at the superior aspect, then cutting the wire's tail leaving 3 knots. The second wire is stitched and wrapped as the same as the first one.
4. The final step is to bend and shorten them the proximal ends of the K-wire using the Qi technique.^[5]^ Then the hook of the K-wire is buried into the quadriceps tendon and hammered into the bone. The distal K-wire ends are cut leaving only 5-mm-length protruding from the distal bone end to prevent retrograde wire migration.

### 2.1 Illustrative case

**Figure 1:**
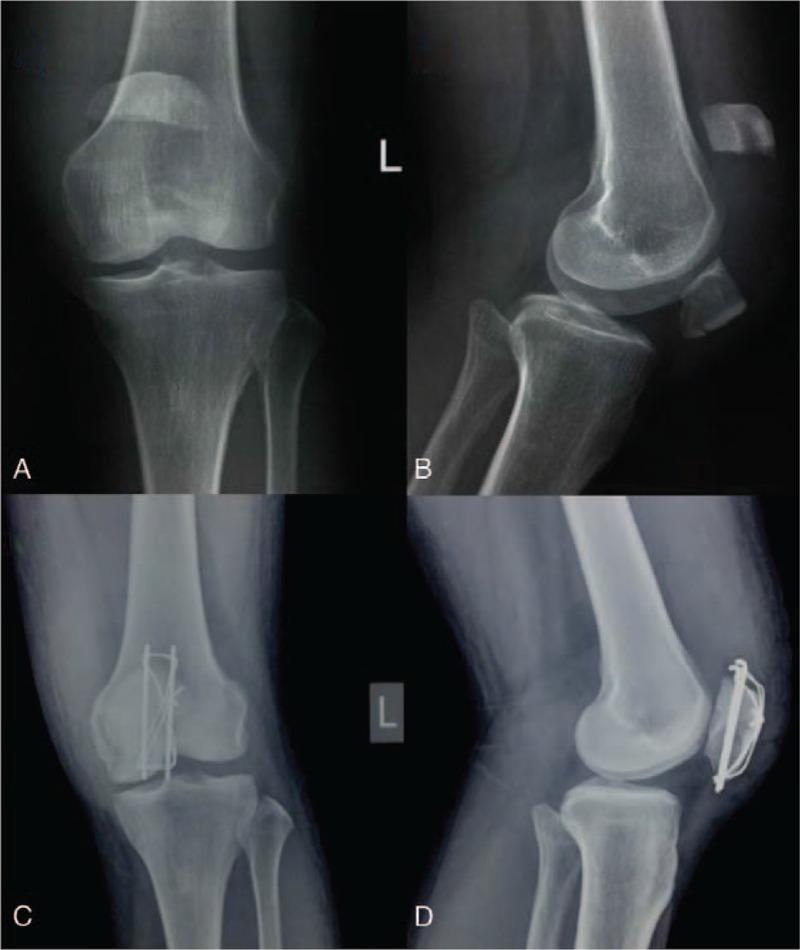
(A) Preoperative anteroposterior and (B) lateral radiographs showing a transverse fracture of the left patella with an inferior pole occult fracture. (C) Postoperative anteroposterior and (D) lateral radiographs showing an anatomic reduction and rigid fixation.

**Figure 2:**
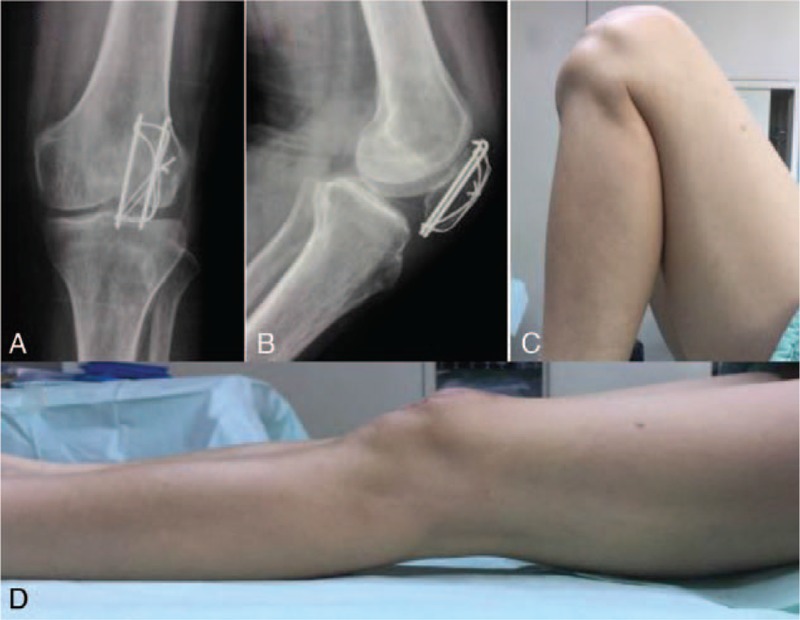
(A) Anteroposterior and (B) lateral radiographs at 1-year postoperatively showed perfect healing of the patella fracture. (C) and (D) Functional photograph of the left knee showing an excellent range of motion of the injury knee.

A 60-year-old man slipped down on the ground and injured his left knee. X-ray obtained showed a transverse fracture of the left patella with an inferior pole occult fracture (Fig. 1A, B). He chose to undergo open reduction and internal fixation with tension band wiring. Operation was carried out with our modified tension band wiring technique. Postoperative x-ray (2 days postop) showed an anatomic reduction and rigid fixation (Fig. 1C, D). Functional exercises were initiated on the third postoperative day. The patient was followed-up for 1 year and the fracture has united very well (Fig. 2A, B), with satisfying knee range of motion (Fig. 2C, D).

Ethical review: The patient's consent has been obtained and the Institutional Review Board of Jilin University approved the publication of this project.

## 3 Discussion

Patellar fractures can be caused by indirect or direct force. The indirect force may result in a transverse fracture when the force exerted exceeds patellar tensile strength. While direct force often results a comminuted fracture.^[6]^ Recent years numerous surgical procedures have been reported; and the most popular surgical technique is tension band wiring due to the biomechanical and economic advantages, which converts the tensile forces produced by the knee extensor to compressive forces at the joint line. The typical tension band wiring technique involves tension wire wrapped around 2 parallel K-wires.^[7]^ This technique is associated with complications such as K-wire migration and tension wire breakage. Several alternative methods have been proposed to prevent this complication such as cerclage wire fixation^[8]^ and plate fixation,^[9]^ but they all have drawbacks especially the cost of the implants.

In our technique, we use a double bundle wire stitching, so when one of the wrapped wires open up or break the second one can hold the reduction and fixation. And we suture the wires under the K-wires and tendons which can prevent the wires to migrate and slip off. Ali et al^[10]^ reported additional strands in the figure-of-8 loop increased the compression and resistance significantly to cyclic loads. So our technique increases the stability of the fracture site which further allows early functional exercise and accelerates fracture union. At the same time, we use the Qi technique to create a standard hook which can hold the wrapped wire in place and prevent its migration.^[5]^ The procedure is simple to perform and no special tools are required without increasing the cost. We have treated over 20 patients with this technique, with no adverse events.

To conclude, our modified tension band wiring of patellar fractures was associated with no implant failure and yielded good clinical outcomes. Based on the above advantages, our technique is worthy of clinical application.

## Author contributions

**Conceptualization:** Xiuxin Liu, Baochang Qi.

**Supervision:** Weina Ju.

**Writing – original draft:** Tiecheng Yu, Zhendong Wu.

**Writing – review & editing:** Sayid Omar Mohamed.
